# Validity and reliability studies of the Indonesian version of Atrial Fibrillation Severity Scale (AFSS)

**DOI:** 10.1186/s12872-023-03240-9

**Published:** 2023-04-28

**Authors:** Muhammad Yamin, Simon Salim, Siti Setiati, Angga Pramudita Pudianto, Putri Zulmiyusrini, Sally Aman Nasution, Ika Prasetya Wijaya, Lusiani Rusdi, Birry Karim, Raden Fidiaji Hiltono Santoso, Friska Anggraini Helena Silitonga

**Affiliations:** 1 Division of Cardiology, Department of Internal Medicine, Faculty of Medicine, Universitas Indonesia – Cipto Mangunkusumo Hospital, Jl. Pangeran Diponegoro No.71, Central Jakarta, Jakarta, Indonesia; 2 Division of Geriatrics, Department of Internal Medicine, Faculty of Medicine, Universitas Indonesia – Cipto Mangunkusumo Hospital, Jakarta, Indonesia; 3 Department of Internal Medicine, Faculty of Medicine, Universitas Indonesia – Cipto Mangunkusumo Hospital, Jakarta, Indonesia

**Keywords:** Atrial fibrillation, Severity, Reliability, Validity, Quality of life

## Abstract

**Background:**

In the atrial fibrillation (AF) population, worsened quality of life (QOL) has been reported even before complications occur. Symptom-based questionnaires can be used to evaluate AF treatment. The Atrial Fibrillation Severity Scale (AFSS) was first developed in Canada in English, which is not the main language in Indonesia. This study aims to test the reliability and validity of the Indonesian version of the Atrial Fibrillation Severity Scale (AFSS).

**Methods:**

Translation of the AFSS from English to Indonesian was done using forward and backward translation. The final version was then validated with the Short Form-36 (SF-36) questionnaire, and a test-retest reliability study was done in a 7-14-day interval.

**Results:**

An Indonesian version of AFSS was achieved and deemed acceptable by a panel of researchers. This version is reliable and valid, with Cronbach’s α of 0.819, Intraclass Correlation Coefficient (ICC) ranging from 0.803 to 0.975, and total score correlation ranging from 0.333 to 0.895. Pearson’s analysis of AFSS and SF-36 revealed that the total AF burden domain was poorly correlated with role limitations due to emotional problems (r:0.427; p < 0.01) and pain (r:0.495; p < 0.01). The symptom severity domain was poorly correlated with physical functioning (r:-0.335; p < 0.01), role limitations due to emotional problems (r:0.499; p < 0.01), pain (r:0.458; p < 0.01), and total SF-36 score (r:-0.361; p < 0.01). Total AFSS score was moderately correlated with role limitations due to emotional problems (r:0.516; p < 0.01) and pain (r:0.538; p < 0.01). The total AFSS score was poorly correlated with the European Heart Rhythm Association (EHRA) score (r:0.315; p < 0.01).

**Conclusion:**

The Indonesian version of AFSS has good internal and external validity with good reliability.

**Supplementary Information:**

The online version contains supplementary material available at 10.1186/s12872-023-03240-9.

## Background

Atrial Fibrillation (AF) the most common sustained supraventricular arrhythmia [1]. The European Society of Cardiology (ESC) reported that the prevalence of AF is between 2 and 4% worldwide and is expected to rise 2.3 times due to population longevity [2]. The Asia Pacific Heart Rhythm Society (APHRS) reported that the 10-year overall incidence in Asia was 1.51–1.77 per 1,000 population. The Asia Pacific prevalence of AF was 0.49–5.4% [3]. AF is associated with higher risk of stroke, peripheral embolism, and ventricular dysfunction. These complications may lead to reduced quality of life (QOL) [4].

The effect of AF on daily life has been well-established by several previous studies. AF patients have higher risk for stroke and heart failure [4, 5]. Even before such complications occur, AF can reduce QOL. It is in the patient’s interest to improve their QOL, not just prevent complications [6].

The main aims of AF management are to reduce symptoms and prevent AF complications, with the hope of maintaining or even improving QOL [7]. Treatment advancement can be evaluated by comparing the scores of simple tools during routine control visits [8]. Aliot et al. reported that the Atrial Fibrillation Severity Scale (AFSS) is one of the most common symptom scales to monitor AF symptoms [7]. In contrast to other questionnaires such as the Symptom Checklist (SCL) that is used to evaluate only symptoms of short duration or the Quality of Life in Atrial Fibrillation (QLAF) that is used to evaluate the impact of AF symptoms in patients, AFSS can be used to evaluate both symptom severity and the impact of these symptoms on patient QOL. Also, the AFSS takes a only short time to fill, so it has a higher response rate compared to longer questionnaires such as SF-36 [9]. Nevertheless, language is a barrier to AFSS use since English is not the mother tongue in Indonesia. AFSS was first developed at the University of Toronto, Canada, hence, cultural barriers should also be considered.

## Methods

This study aims to test the reliability and validity of the Indonesian version of AFSS. This cross-sectional study was conducted in March - April 2022. The subjects were AF patients aged ≥ 18 years with a high degree of fluency in the Indonesian language. The exclusion criteria of this study were history of hospitalization due to acute or severe chronic conditions within the previous month, history of cardiac surgery within the previous month, physical handicaps, or mental or psychiatric disorders.

General characteristics such as gender, age, highest education level attained, and marital status were taken from medical records, while European Heart Rhythm Association (EHRA) score and duration of diagnosis were obtained during history-taking. Subjects’ ejection fraction (EF) were measured within the previous 3 months by echocardiography examination.

Participants were encouraged to independently fill the AFSS and Indonesian version of the SF-36 (gold standard) questionnaires [10]. A second round of testing was done 7–14 days after the first test. This study was approved by the Health Research Ethics Committee, Faculty of Medicine Universitas Indonesia, Cipto Mangunkusomo Hospital, with approval number KET-/054/UN2.F1/ETIK/PPM.00.02/2021.

### Translation

The translation process was conducted in two phases after obtaining permission from the original authors. For the first phase, forward translation from English to Indonesian was done by two translators, one medical and one non-medical. Both were certified and experienced as translators and interpreters. After translating the AFSS independently from each other, they discussed and combined their translations into the Indonesian Synthesis Translation. This version underwent a backward translation (Indonesian to English) by two native English speakers, who both had nearly ten years of experience in interpreting and translating English to Indonesian and vice versa. Both backward translations were then compared to the original version of AFSS, as seen in Supplementary 1. Any cultural adaptations and differences were reviewed by the research team before the pre-final questionnaire was distributed to participants. This version of the questionnaire was first pretested on 30 participants, then edited based on patient feedback, forming the final version of the questionnaire, which was then used for the second phase of testing.

### Questionnaire

The AFSS is a disease-specific questionnaire to assess AF severity and note the symptom changes over time. It consists of 19 questions, divided into 3 parts. Part A consists of 8 questions concerning general characteristics, as well as AF frequency and severity. Part B consists of 4 questions concerning the history of cardioversion, hospitalization, emergency room visits, and specialist appointments due to symptomatic AF in the previous year. Part C consists of 7 questions concerning AF severity in the previous 4 weeks. Scores range from 0 to 5, with higher scores indicating worse symptoms [7].

### Data analysis

The data recorded were analyzed using SPSS Statistics 26.0 software and are presented in tables. General characteristics are presented as frequency and percentage. Kolmogorov-Smirnov test was used to assess data normality (n > 50). Normally distributed data (p > 0.05) were evaluated with Pearson’s test and non-normally distributed data (p < 0.05) were evaluated with Spearman’s test. AFSS score was first converted to a 0-100 scale and reversed as needed to balance the scores between questions.

Test-retest reliability analysis resulted in Cronbach’s α and Intraclass Correlation Coefficient (ICC) between the first test and the retest. For test validity, Cronbach’s α values were interpreted as low (< 0.6), acceptable (0.6–0.8), or very good internal consistency (> 0.8) [11]. ICC values were classified as poor (< 0.5), moderate (0.5-<0.75), good (0.75-<0.9), or excellent (> 0.9) reliability [12]. Validity was established by bivariate correlation analysis by evaluating the inter-item correlation and total score.

We also analyzed the AFSS domains, SF-36, and clinical parameters. The degrees of correlation were classified as very low (r:0.01–0.2), low (r:0.21–0.4), moderate (r:0.41–0.6), strong (r:0.61–0.8), or very strong (r:0.81–0.99) [13].

## Results

### General characteristics

Sixty participants were recruited for the second phase of testing the final version of the AFSS. Subjects’ general and clinical characteristics are shown in Table 1. Most participants were male (53.3%), and the majority age group was 61–70 years (31.6%). The mean age of our participants was 58.8 years. Most participants were classified as EHRA IIa (40%).

Most subjects had EF ≥ 50%, with a mean of 56.8%. Heart failure was reported in 75% of participants. More than half of participants had valvular disorders (53.3%), while 78.3% had tricuspid regurgitation, and 75% had mitral disorders (76.7% mitral regurgitation and 35% mitral stenosis). The AFSS retest took a shorter time to fill than the initial test. A descriptive analysis of general characteristics is shown in Supplementary 2.

**Table 1:** General and Clinical Characteristics of Participants

Characteristics	(N = 60)	%
**Gender**		
Male	32	53.3
Female	28	46.7
**Age, years**		
≤ 40	5	8.3
41–50	8	13.3
51–60	17	28.4
61–70	19	31.7
> 70	11	18.3
**Education level attained**		
Primary or less	6	10
Junior high	6	10
Senior high	22	36.7
University	26	43.3
**Marital status**		
Single	6	10
Married	50	83.3
Widow/Widower	4	6.7
**EHRA**		
I	4	6.7
IIa	24	40
IIb	18	30
III	12	20
IV	2	3.3
**Ejection fraction, %**		
< 40	5	8.3
40–49	9	15
≥ 50	46	76.7
**Duration of diagnosis, years**		
< 1	2	3.3
1–5	50	83.4
> 5	8	13.3
**AF etiology**		
Valvular	32	53.3
Non-valvular	28	46.7
**AF type**		
Paroxysmal	11	18.3
Persistent	9	15
Longstanding persistent	2	3.3
Permanent	36	63.3
**Duration to complete (minute)**		
Test		
Mean, SD	8.8 (2.1)	
Range	5–14	
Retest		
Mean, SD	8.1 (1.7)	
Range	5–12	

### Indonesian version of AFSS Reliability and Validity

The power (1-β) analysis in our study was performed using G*Power software. The test used was in the T test family, correlation: point biserial model as the statistical test and post hoc as the type of power analysis. After calculation, almost all of the significant values had a power of at least 80% in all variables, which is acceptable for medical research.

Our Indonesian version of AFSS was acceptable, as shown by Cronbach’s α > 0.6 and ICC score (r > 0.330). Cronbach’s α of the Indonesian version of AFSS was 0.819, which was considered to be excellent internal consistency. Cronbach’s α domain values were as follows: total AF burden domain 0.651, health care utilization domain 0.713, and symptom severity domain 0.778.

The ICC reflects the correlation between the test and retest. The ICC domain scores were as follows: total AF burden domain 0.956, health care utilization domain 0.961, and symptom severity domain 0.948, all indicating excellent reliability. In our study, the ICC values ranged from 0.803 to 0.975, which indicated good or excellent reliability. Our version of AFSS also had good reliability (p < 0.01), with total score correlation ranging from 0.333 to 0.895. The reliability and validation analyses are shown in Table 2.

The total AF burden domain was poorly correlated with health care utilization (r:0.282; p < 0.05), moderately correlated with symptom severity (r:0.627; p < 0.01), and well correlated with total AFSS score (r:0.895; p < 0.01). The health care utilization domain was weakly correlated with symptom severity (r:0.365; p < 0.01) and moderately correlated with total AFSS score (r:0.508; p < 0.01). The symptom severity domain was very well correlated with total AFSS score (r:0.880; p < 0.01).

**Table 2:** Reliability and Validity Analysis of the Indonesian Version of AFSS

Variables	Correlation				ICC D_1_ and D_8 − 14_	Cronbach α
	Total AF Burden Domain	Health Care Utilization Domain	Symptom Severity Domain	Total		
**Total AF Burden Domain**	-	0.282*	0.627**	0.895**	0.956**	0.651
A3 A4 A5 A6 A7 A8	0.404** 0.477** 0.812** 0.550** 0.754** 0.617**	0.213 0.055 0.294* 0.365** 0.095 0.078	0.224 0.244 0.547** 0.414** 0.431** 0.252	0.361** 0.443** 0.756** 0.561** 0.666** 0.470**	0.803** 0.918** 0.904** 0.939** 0.926** 0.911**	
**Health Care Utilization Domain**	0.282*	-	0.365**	0.508**	0.961**	0.713
B9 B10 B11 B12	0.315* 0.265* 0.254 0.128	0.397** 0.920** 0.909** 0.594**	0.270* 0.243 0.319* 0.319*	0.374** 0.440** 0.442** 0.333*	0.808** 0.958** 0.963** 0.846**	
**Symptom Severity Domain**	0.627**	0.365**	-	0.880**	0.948**	0.778
C1 C2 C3 C4 C5 C6 C7	0.464** 0.403** 0.311* 0.490** 0.221 0.424** 0.541**	0.474** 0.399** 0.403** 0.033 0.106 0.071 0.233	0.693** 0.736** 0.644** 0.641** 0.688** 0.618** 0.607**	0.660** 0.638** 0.538** 0.564** 0.487** 0.525** 0.615**	0.961** 0.895** 0.919** 0.888** 0.942** 0.975** 0.945**	
**Total**	0.895**	0.508**	0.880**	-	0.963**	0.819

* Significant correlation in α = 0.05 (2-tailed)

** Significant correlation in α = 0.01 (2-tailed)

### The correlation between Indonesian version of AFSS and SF-36

In comparison to SF-36, the total AF burden domain was poorly correlated with role limitations due to emotional problems (r:0.427; p < 0.01), poorly correlated with fatigue (r:-0.326; p < 0.05), and poorly correlated with pain (r:0.495; p < 0.01). The health care utilization domain was poorly correlated with role limitations due to emotional problems (r:0.279; p < 0.05). The symptom severity domain was poorly correlated with physical functioning (r:-0.335; p < 0.01), role limitations due to physical health (r:0.321; p < 0.05), role limitations due to emotional problems (r:0.499; p < 0.01), social functioning (r:-0.282; p < 0.05), pain (r:0.458; p < 0.01), general health (r:0.270; p < 0.05), and total SF-36 score (r:-0.361; p < 0.01). Total AFSS score was moderately correlated with role limitations due to emotional problems (r:0.516; p < 0.01), moderately correlated with pain (r:0.538; p < 0.01), poorly correlated with general health (r:0.274; p < 0.05), and poorly correlated with total SF-36 score (r:-0.378; p < 0.05), as shown in Table 3. Descriptive analysis of the AFSS score can be seen in Supplementary 3.

**Table 3:** Correlation between the Indonesian version of AFSS and SF-36

SF-36 Domain	Total AF Burden Domain	Health Care Utilization Domain	Symptom Severity Domain	Total AFSS Score
Physical functioning	-0.041	0.002	-0.335**	-0.175
Role limitations due to physical health	0.160	0.155	0.321*	0.207
Role limitations due to emotional problems	0.427**	0.279*	0.499**	0.516**
Energy/fatigue	-0.326*	-0.070	-0.041	-0.218
Emotional well-being	-0.107	-0.042	-0.015	-0.009
Social functioning	-0.087	-0.065	-0.282*	-0.168
Pain	0.495**	0.234	0.458**	0.538**
General health	0.110	0.172	0.270*	0.274*
Total score	-0.090	0.117	-0.361**	-0.387**

All data were analyzed using Pearson’s test

* Significant correlation in α = 0.05 (2-tailed)

** Significant correlation in α = 0.01 (2-tailed)

### The correlation between Indonesian Version of AFSS, Ejection Fraction, and EHRA

In our study, total AF burden, health care utilization, symptom severity, and total AFSS score were not correlated with EF. In addition, total AF burden, health care utilization, and symptom severity domain were not correlated with EHRA. However, the total AFSS score was poorly correlated with the EHRA score (r:0.315; p < 0.05), as shown in Table 4.

**Table 4:** Correlation between Indonesian Version of AFSS and Other Clinical Parameters

Parameters	Total AF Burden Domain	Health Care Utilization Domain	Symptom Severity Domain	Total AFSS
EF	0.107	0.72	-0.2	-0.006
EHRA	0.179	0.179	0.033	0.315*

All data were analyzed using Pearson’s test

* Significant correlation in α = 0.05 (2-tailed)

** Significant correlation in α = 0.01 (2-tailed)

## Discussion

Jones et al. reported that AF patients had lower QOL compared to the general population and those with other cardiovascular diseases. AF symptoms such as tachycardia, shortness of breath, chest pain, sleeping difficulties, and psychological distress contribute to worse QOL in AF patients [6]. The QOL reduction in AF patients was even reported to be comparable to patients with postmyocardial infarction. QOL is often assessed with standardized questionnaires that have been adapted to local cultures [14].

The Indonesian version of AFSS revealed good internal consistency, based on the high Cronbach’s α value (0.819) and ICC score (0.963). This AFSS version was also found to be valid, as all total correlations scored > 0.330. The overall score of AFSS and SF-36 showed a poorly negative correlation (r:-0.387; p < 0.01), meaning that higher AFSS score (highly symptomatic) was correlated with lower SF-36 score (worse QOL). The SF-36 total score also showed a poorly negative correlation with the symptom severity domain (r:-0.361; p < 0.01), which means that higher symptom severity score (more severe AF symptoms) was correlated with lower SF-36 score (worse QOL). The total score of the Indonesian version of AFSS showed strong correlations (r > 0.60) with SF-36, as also seen in the Turkish version of AFSS. However, the lack of strong correlations among the domains of the two tools is evidence that they could be used to complement each other in patient HRQOL evaluations [14].

The ICC for the various domains ranged from 0.803 to 0.975, which were considered to have good or excellent reliability. This finding indicates that in the 7-14-day interval between testing, there were no major changes in the symptoms of AF patients. Lindberg et al. stated that AF is a chronic lifelong condition which requires regular medical control [15]. Heidt et al. reported that several validated AF questionnaires such as AF-QoL, AFEQT, and ASTA are recommended for use every 1–3 months [16]. In our study, AF patients with recent acute or severe chronic conditions and cardiac surgery were excluded to minimize bias of rapidly improved symptoms.

Although AF is not considered to be immediately life-threatening, it is a chronic condition that significantly decreases QOL in patients. Dorian et al. reported that worse symptom severity significantly affected the physical and emotional components of QOL, general well-being, and health care utilization [17]. This result was in agreement with our study, in which higher symptom severity score was poorly correlated with worse physical functioning (r:-0.335; p < 0.01), was poorly correlated with worse pain (r:0.458; p < 0.01), and was moderately correlated with limitation of daily activities due to emotional problems (r:0.516; p < 0.01).

Bodily pain had a poor correlation with total AF burden (r:0.495; p < 0.01), symptom severity (r:0.458; p < 0.01), and moderately correlation with total AFSS score (r:0.538; p < 0.01) in this study. A similar result was reported by Eren et al., who noted that bodily pain was poorly correlated with global well-being (r:0.24; p < 0.01) and total AF burden (r:-0.26; p < 0.01), and moderately correlated with symptoms domain (r:-0.58; p < 0.01) in AFSS [14]. However, the majority of participants reported knee pain (53.33%) and low back pain (38.33%), instead of cardiac chest pain. Wong et al. reported that musculoskeletal complaints ranged from 65 to 85% in the elderly and increased with older age. Knee pain and low back pain in the elderly are often related to degenerative processes such as osteoarthritis, lumbar disc degeneration, and osteoporotic fractures [18, 19].

Physical limitations in AF are related to level of fatigue, shortness of breath, and underlying causes of AF [20]. Atrial contraction could contribute up to 20% of stroke volume at rest. However, this contribution is lost during AF episodes. This results in lower coronary flow and left ventricular dysfunction, which contribute to fatigue and dyspnea development [21]. Even though AF was poorly correlated with physical functioning (r:-0.335; p < 0.01) and role limitations due to physical health (r:0.321; p < 0.05), we found no correlation between the severity of AF symptoms and EHRA classification. This discrepancy was in agreement with a study by Wynn et al., who reported that EHRA score had no significant discriminatory power in EHRA class 1 and 2a as compared to QOL, and QOL reduction only started from class 2b [22]. In our study, 46.67% of participants were classified as EHRA class 1 and 2b. Hence, a proper assessment such as with the AFSS questionnaire is required to examine an absolute physical influence of AF.

Physiological distress in AF has long been established. Anxiety (35%) and depression (20%) was found in patients with permanent AF. This distress is related to long-term medication use and side effects, fear of worsening symptoms, symptoms emerging during activity, and interventions planned to control symptoms [20]. Otherwise, physiological distress may also induce AF development by stimulating sympatho-vagal activation and autonomic ganglia [23].

In our study, total AF burden (r:0.427; p < 0.01) and symptom severity domain (r:0.499; p < 0.01) had a poor correlation with role limitations due to emotional problems. Approximately 50% of participants reported fear of arrhythmia exacerbation during daily activities, which leads to role limitations due to anxiety. However, none of the AFSS domains were significantly correlated with emotional well-being in SF-36. This finding was similar to a Turkish study, which reported that global well-being (r:0.33; p < 0.01), total AF burden (r:0.32; p < 0.01), and symptom severity domain (r:0.53; p < 0.01) were correlated with role limitations due to emotional problems [14].

Sharma reported that good questionnaires should be able to be administered within 30 min and questionnaires with more than 30 questions should be done on divided occasions to keep participants’ full attention. Longer questionnaires have been correlated with more missing data and non-responsive participants [24]. In our study, participants only required 8.8 min for the initial test and 8.1 min for the retest). This duration was shorter than that of the Atrial Fibrillation Effect on QualiTy-of-life (AFEQT) test (9.3 min) and SF-36 (> 15 min) [9]. Thus, filling the AFSS is feasible, especially during a control appointment of a short duration time. A limitation of our study was that the small sample size might have affected the degree of statistical significance.

## Conclusion

The Indonesian version of AFSS has good internal and external validity and good reliability to evaluate AF severity. This scale can be used to compare symptom development before and after therapy. Higher total AF burden, symptom severity, and AFSS total score are correlated with limitation of daily activities due to emotional problems, while symptom severity itself is strongly correlated with physical functioning of patients with AF.

## Electronic supplementary material

Below is the link to the electronic supplementary material.

Supplementary Material 1: Questionnaire Translation Process and AFSS Descriptive Analysis

## Data Availability

The datasets used and/or analyzed during our study are available from the corresponding author on reasonable request.

## Abbreviations

AF: Atrial Fibrillation
AFSS: Atrial Fibrillation Severity Scale
APHRS: Asia Pacific Heart Rhythm Society
EF: Ejection Fraction
EHRA: European Heart Rhythm Association
ESC: European Society of Cardiology
ICC: Intraclass Correlation Coefficient
QOL: Quality of Life
