# Supplementary material for: Validity and reliability studies of the Indonesian version of Atrial Fibrillation Severity Scale (AFSS)
Source: BMC Cardiovasc Disord. 2023 Apr 28;23:216. doi: 10.1186/s12872-023-03240-9 (PMC10148504; doi:10.1186/s12872-023-03240-9)
Supplement: Supplementary file 1 — Supplementary Material 1: Questionnaire Translation Process and AFSS Descriptive Analysis [file 12872_2023_3240_MOESM1_ESM.docx]

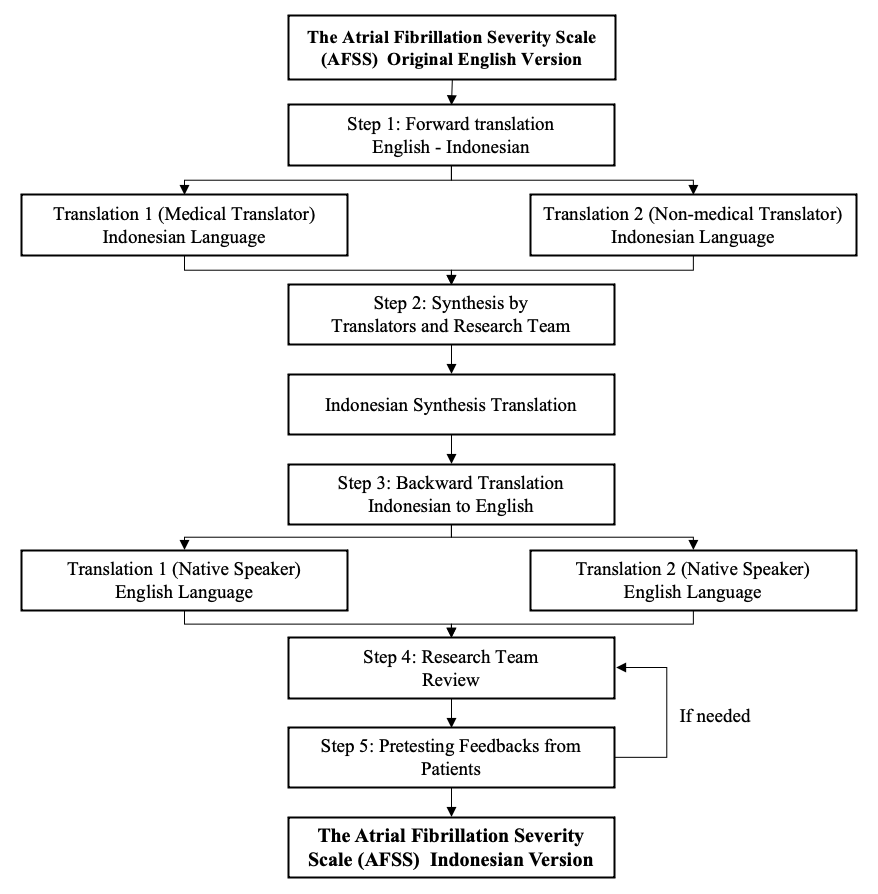
Supplementary 1 Questionnaire Translation Process

Supplementary 2 Characteristics of AF Patients

| **Characteristics** | **n=60** | **%** |
| --- | --- | --- |
| **Age** |  |  |
| <40 | 5 | 8.3 |
| 41-50 | 8 | 13.3 |
| 51-60 | 17 | 28.4 |
| 61-70 | 19 | 31.7 |
| >70 | 11 | 18.3 |
| Range | 20-83 | |
| Average (SD) | 58.8 (13.6) | |
| **Duration of Diagnosis** |  |  |
| <1 | 2 | 3.3 |
| 1-5 | 50 | 83.3 |
| >5 | 8 | 13.3 |
| Range | 1-27 | |
| Average (SD) | 4.3 (4.5) | |
| **Ejection Fraction (%)** |  |  |
| <40 | 5 | 8.3 |
| 40-49 | 9 | 15.0 |
| >50 | 46 | 76.7 |
| Range | 23-75.9 | |
| Average (SD) | 56.8 (10.8) | |
| **CHA2DS2VASC Score** |  |  |
| 0 | 0 | 0.0 |
| 1 | 4 | 6.7 |
| >1 | 56 | 93.3 |
| Range | 1-7 | |
| Average (SD) | 3.2 (1.5) | |
| **HASBLED** |  |  |
| 0 | 22 | 48.3 |
| 1-2 | 36 | 50.0 |
| >3 | 2 | 1.7 |
| Range | 0-5 | |
| Average (SD) | 1.1 (1.1) | |

Supplementary 3 AFSS Score Descriptive Analysis

| **AFSS Category** | **Min** | **Max** | **Average** | **SD** |
| --- | --- | --- | --- | --- |
| **AF Burden** |  |  |  |  |
| *Day 1* |  |  |  |  |
| Life satisfaction | 2 | 11 | 7.13 | 1.58 |
| AF Frequency | 1 | 12 | 6.80 | 3.17 |
| AF duration | 1 | 11 | 6.98 | 1.83 |
| Severity of the last AF period | 1 | 10 | 4.53 | 2.23 |
| Severity of the first AF period | 1 | 10 | 5.70 | 2.61 |
| *Day 8-14* |  |  |  |  |
| Life satisfaction | 2 | 11 | 7.08 | 1.52 |
| AF Frequency | 1 | 12 | 6.42 | 3.15 |
| AF duration | 1 | 12 | 6.83 | 2.00 |
| Severity of the last AF period | 1 | 10 | 4.40 | 2.12 |
| Severity of the first AF period | 1 | 10 | 5.47 | 2.85 |
| **Health Care Utilization** |  |  |  |  |
| *Day 1* |  |  |  |  |
| Cardioversion (times) | 0 | 6 | 1.90 | 0.71 |
| ER visit (times in a year) | 0 | 5 | 0.50 | 1.07 |
| Hospitalization (times in a year) | 0 | 5 | 0.55 | 0.96 |
| Outpatient visit (times in a year) | 0 | 3 | 0.37 | 0.76 |
| *Day 8-14* |  |  |  |  |
| Cardioversion (times) | 0 | 6 | 1.92 | 0.59 |
| ER visit (times in a year) | 0 | 5 | 0.58 | 1.06 |
| Hospitalization (times in a year) | 0 | 5 | 0.62 | 0.96 |
| Outpatient visit (times in a year) | 0 | 3 | 0.33 | 0.66 |
| **Symptom Severity** |  |  |  |  |
| *Day 1* |  |  |  |  |
| Palpitation | 0 | 5 | 1.27 | 1.27 |
| Shortness of breath at rest | 0 | 4 | 0.82 | 1.24 |
| Shortness of breath during physical activity | 0 | 5 | 1.52 | 1.51 |
| Fatigue during light physical activity | 0 | 5 | 1.97 | 1.53 |
| Fatigue at rest | 0 | 4 | 0.95 | 1.35 |
| Lightheaded/dizzy | 0 | 5 | 1.58 | 1.57 |
| Pain or pressure in the chest | 0 | 5 | 1.30 | 1.48 |
| Total | 0 | 27 | 9.40 | 6.54 |
| *Day 8-14* |  |  |  |  |
| Palpitation | 0 | 5 | 1.30 | 1.21 |
| Shortness of breath at rest | 0 | 4 | 0.77 | 1.17 |
| Shortness of breath during physical activity | 0 | 5 | 1.40 | 1.43 |
| Fatigue during light physical activity | 0 | 5 | 2.13 | 1.40 |
| Fatigue at rest | 0 | 4 | 0.98 | 1.24 |
| Lightheaded/dizzy | 0 | 5 | 1.60 | 1.62 |
| Pain or pressure in the chest | 0 | 5 | 1.28 | 1.47 |
| Total | 0 | 27 | 9.47 | 6.25 |
